# Poly (A) Binding Protein Cytoplasmic 1 Is a Novel Co-Regulator of the Androgen Receptor

**DOI:** 10.1371/journal.pone.0128495

**Published:** 2015-07-15

**Authors:** Kurtis Eisermann, Javid A. Dar, Jun Dong, Dan Wang, Khalid Z. Masoodi, Zhou Wang

**Affiliations:** 1 Department of Urology, University of Pittsburgh School of Medicine, Pittsburgh, Pennsylvania, United States of America; 2 Department of Pharmacology and Chemical Biology, University of Pittsburgh School of Medicine, Pittsburgh, Pennsylvania, United States of America; 3 University of Pittsburgh Cancer Institute, University of Pittsburgh School of Medicine, Pittsburgh, Pennsylvania, United States of America; 4 Central Laboratory College of Science, King Saud University, Riyadh, Saudi Arabia; 5 Division of Biotechnology, Sher-e-Kashmir University of Agricultural Sciences and Technology, Shalimar, Srinagar, J&K, India; Hormel Institute, University of Minnesota, UNITED STATES

## Abstract

The androgen receptor (AR) is a member of the steroid receptor superfamily that regulates gene expression in a ligand-dependent manner. The NTD of the AR plays a key role in AR transactivation including androgen-independent activation of the AR in castration-resistant prostate cancer (CRPC) cells. We recently reported that amino acids (a.a.) 50-250 of the NTD are capable of modulating AR nucleocytoplasmic trafficking. To further explore the mechanism associated with a.a. 50-250, GFP pull-down assays were performed in C4-2 CRPC cells transfected with GFP tagged a.a. 50-250 of the AR. Mass spectrometry analysis of the pulled down proteins identified poly (A) binding protein cytoplasmic 1 (PABPC1) interaction with this region of the AR. *In silico *analysis of gene expression data revealed PABPC1 up-regulation in prostate cancer tissue specimens and this up-regulation correlates to increased disease recurrence. Co-immunoprecipitation assays confirmed the association of PABPC1 with a.a. 50-250 of the NTD of the AR. Knockdown of PABPC1 decreased nuclear AR protein levels and inhibited androgen activation of the AR target PSA in LNCaP and C4-2 cells. Additionally, knockdown of PABPC1 inhibited transactivation of the PSA promoter by NAR (AR lacking the LBD) and attenuated proliferation of AR-positive prostate cancer cells. These findings suggest that PABPC1 is a novel co-regulator of the AR and may be a potential target for blocking activation of the AR in CRPC.

## Introduction

Prostate cancer is the second leading cause of cancer death in the United States killing almost 30,000 men annually [1]. Localized prostate cancer that is confined entirely to the prostate gland is curable, but when the cancer has spread to distant tissues, organs, and lymph nodes, the five year survival rate is only 28% [1]. Since androgens are known to participate in the progression of prostate cancer, the first line of therapy for patients with metastatic prostate cancer is usually androgen deprivation therapy (ADT). ADT involves using drugs such as lupron and bicalutamide that target androgen receptor (AR) signaling through lowering serum testosterone or blocking the binding of androgens to the AR [2–6]. However, most patients eventually relapse and the cancer becomes resistant to hormone therapy, a condition known as castration-resistant or–recurrent prostate cancer (CRPC). Abiraterone and enzalutamide are newly approved drugs for CRPC patients; although, patients will still eventually relapse and the overall survival is prolonged only ~4–5 months after treatment with the AR signaling becoming active in most of these relapsed tumors [7,8]. Therefore, a detailed understanding of the mechanisms regulating AR function may lead to novel approaches targeting the AR in CRPC as well as in CRPC relapsed after treatment with abiraterone and enzalutamide.

The AR is a member of the steroid receptor superfamily that regulates gene expression in a ligand-dependent manner. It plays a vital role in all stages of prostate cancer development and progression, including CRPC. The N-terminal domain (NTD) of the AR is the least conserved of the four domains of the receptor and does not have an ordered structure [9,10]. It contains the activation function 1 (AF1) element that is crucial for AR transactivational activity [11]. AF1 is located between amino acids 101 and 370 and is required for full ligand activated transcriptional activity. Additionally, activation function 5 (AF5) which is located between residues 360–485 is responsible for AR constitutive activity in the absence of ligand [11]. The NTD of the AR plays a key role in AR transactivation including androgen-independent activation of the AR in CRPC cells. Previous studies by our group determined that the NTD of the AR contains a region, a.a. 50–250, that can regulate its nucleocytoplasmic localization [12]. However, the mechanism by which the a.a. 50–250 region modulates AR subcellular localization is not clear. Binding partners of this region are likely to play a role in modulating AR subcellular localization and function. Thus, identification of binding partners of a.a. 50–250 of the NTD will be crucial to mechanistic studies associated with this region, which may lead to the design of novel therapies.

In the present study, we explored potential mechanisms involved in the modulation of AR subcellular localization and function by the a.a. 50–250 region of the NTD using immunoprecipitation coupled with proteomics. This led to the identification of poly (A) binding protein cytoplasmic 1 (PABPC1) as a binding partner of the region in the NTD capable of promoting AR cytoplasmic localization. PABPC1 is known to bind to the 3’ poly (A) tail of eukaryotic mRNA via RNA recognition motifs [13]. It is involved in shortening of the poly (A) tail and translation initiation [13], and is capable of shuttling between the nucleus and the cytoplasm [14]. Further functional characterization suggests that PABPC1 plays an important role in modulating AR nucleocytoplasmic localization and function in prostate cancer cells.

## Methods

### Cell lines and plasmids

LNCaP, 22Rv1, and PC3 cells were purchased from American Type Culture Collection (Manassas, VA). C4-2 cells were obtained from Dr. Leland Chung (Cedars Sinai Medical Center, Los Angeles, CA). All cells were maintained in RPMI 1640 medium supplemented with 10% fetal bovine serum (FBS), 1% L-glutamine, 100 units/ml penicillin, and 100 μg/ml streptomycin (Invitrogen, Grand Island, NY). Cells were grown at 37°C in the presence of 5% CO_2_ in a humidified incubator. For androgen induction, cells were grown in 5% charcoal stripped FBS RPMI for 24 hours followed by addition of R1881 (Perkin Elmer, Waltham, MA) for an additional 24 hours.

The expression vector pEGFP-C1 (Clontech, Mountain View, CA) was used to generate fusion protein constructs with GFP at the N terminus of AR and various AR mutants as described [12]. The PSA6.1 luciferase reporter construct was generously provided by Dr. Marianne Sadar [15]. The pRL-CMV renilla luciferase reporter construct was purchased from Promega (Sunnyvale, CA).

### GFP pull-down assay

C4-2 cells were transiently transfected with indicated AR deletion constructs tagged with GFP using Polyjet transfection reagent (SignaGen Laboratories, Rockville, MD) in 10% FBS RPMI media. Cells were lysed with NP-40 lysis buffer 48 hours after transfection. GFP pull-down assays were performed using anti-GFP tagged agarose beads (20μl) (MBL International, Woburn, MA) followed by SDS-PAGE. SDS-PAGE gels were stained with coomassie blue to visualize banding patterns in immunoprecipitation compared with GFP control immunoprecipitation. Gel bands that were uniquely pulled down by a.a. 50–250 of the NTD of the AR (GFP-AR^50-250^) were excised and sent for analysis by mass spectrometry at the Cancer Biomarkers Facility (CBF) of University of Pittsburgh Cancer Institute (UPCI) (Pittsburgh, PA).

### Mass spectrometry

Gel bands were excised and destained, disulfides were reduced with Tris (2-carboxyethyl) phosphine hydrochloride (TCEP) (Sigma Aldrich, St Louis, MO), and sulfhydryls were alkylated with iodoacetamide (Sigma Aldrich, St Louis, MO) as previously described [16]. Gel plugs were digested overnight with 13 ng/μl trypsin (Promega, Madison, WI) at 37^°^C. Peptides were extracted with 1:2 (vol/vol) 5% formic acid/acetonitrile for mass spectrometric analysis. MALDI TOF/TOF analysis was done on a 4800 MALDI-TOF/TOF analyzer (ABSciex, Foster City, CA). MALDI TOF/TOF data was analyzed for protein identification with ABSciex Protein Pilot 3.0 using Mascot (version 2.1.0). Searches used the IPI_human_3.68 human (87061 sequences; 35160944 residues) protein database. The search parameters used trypsin as the proteolytic enzyme with one missed cleavage permitted, carbamidomethylation of cysteines and oxidation of methionines as variable modifications, and mass tolerance of 50ppm and 0.4 Da for precursor ions and fragment ions, respectively. Protein score is -10*Log(P), where P is the probability that the observed match is a random event. Protein scores were derived from ion scores as a non-probabilistic basis for ranking protein hits. Protein scores greater than 62 were considered to be significant (p<0.05).

### Co-immunoprecipitation

C4-2 cells were transiently transfected with AR deletion constructs tagged with GFP using Polyjet transfection reagent (SignaGen Laboratories, Rockville, MD). C4-2 cells were also stably transfected with GFP or GFP-tagged AR deletion constructs in the presence of G418. GFP pull-down assays were performed using anti-GFP tagged agarose beads (MBL International, Woburn, MA) followed by Western blot analysis using antibodies specific for PABPC1 (F-2) (Santa Cruz Biotechnology, Dallas, TX) and GFP (Torrey Pines Biolabs, Inc., Houston, TX).

### siRNA knockdown

Control siRNA (sc-37007) and siRNA oligos specific for PABPC1 (sc-108012) were purchased from Santa Cruz Biotechnology (Dallas, TX) and transfected for knock down of PABPC1. Cells were transfected in OPTI-MEM media (Life Technologies, Grand Island, NY) using Lipofectamine 2000 (Life Technologies, Grand Island, NY) according to the manufacturer’s instructions and harvested 72 hours after transfection in RIPA buffer for analysis by Western blot using antibodies specific for PABPC1 (F-2), PSA (C-19) and GAPDH (FL-335) (all purchased from Santa Cruz Biotechnology, Dallas, TX). GAPDH was included as a loading control. For localization of the AR, C4-2 cells were seeded into 10cm^2^ dishes followed by knockdown of PABPC1. Nuclear and cytoplasmic fractions were prepared using the NE-PER Nuclear and Cytoplasmic Extraction kit from Thermo Fisher Scientific following the manufacturer’s recommendations (Rockford, IL). GAPDH and Lamin A/C (Genscript, Piscataway, NJ) were used as loading controls. For mRNA expression following knockdown, cells were assayed as described below. For luciferase assay, cells were transfected with appropriate reporter plasmids and assayed as described below.

### RNA isolation and quantitative real-time PCR

C4-2 and LNCaP cells were cultured in 5% charcoal stripped FBS RPMI for 24 hours and then transfected with either siControl (40 pmol/mL) or siPABPC1 (40 pmol/mL) for 48 hours followed by treatment with DMSO or 0.1nM R1881 for an additional 24 hours. RNA was isolated from subconfluent cells using Trizol (Life Technologies, Carlsbad, CA) followed by phenol chloroform extraction. Following quantitation, 1μg of RNA was reverse transcribed using the High Capacity cDNA Reverse Transcription Kit (Applied Biosystems, Carlsbad, CA). qRT-PCR was performed using Taqman Universal Master Mix (Applied Biosystems, Carlsbad, CA) with either Taqman primers specific for PABPC1 (Hs01598422_mH) or primers/probes spanning exon/exon junctions designed using Primer 3 software (Primer 3, Totowa, NJ): GAPDH For: 5’-CAT GTT CGT CAT GGG TGT GA-3’; Rev: 5’-GGT GCT AAG CAG TTG GTG GT-3’; Probe: 5’-6FAM ACA GCC TCA AGA TCA TCA GCA ATG CCT C TAMRA-3’ and PSA For: 5’-CAT CAG GAA CAA AAG CGT GA-3’; Rev: 5’-AGC TGT GGC TGA CCT GAA AT-3’; Probe: 5’-6FAM CAC AGC CTG TTT CAT CCT GAA GAC ACA TAMRA-3’. The comparative Ct method [17] was used to analyze gene expression differences between control (untreated) cells and cells treated with R1881.

### BrdU cell proliferation assay

C4-2, LNCaP, PC3, and 22Rv1 cells were transfected with siPABPC1 (40 pmol/mL) or control siRNA (40 pmol/mL) for 72 hours followed by addition of BrdU (Sigma Aldrich, St Louis, MO) for 1 hour at 37°C. Cells were then fixed with Carnoy’s fixative (3 vol methanol + 1 vol glacial acetic acid), incubated with 2M HCl, washed with 0.1M boric acid, followed by incubation with 3% H_2_0_2_. After blocking with 10% goat serum, cells were incubated with anti-BrdU antibody (Sigma Aldrich, St Louis, MO) overnight, followed by addition of Cy3 secondary antibody (Life Technologies, Grand Island, NY). Cells were visualized with a Nikon Eclipse TE 2000-U fluorescence microscope (Nikon Instruments, Inc., Melville, NY). Images were taken at 100x total magnification and all BrdU positive cells were counted in each image. The mean average of three experiments from cells transfected with control siRNA was compared to cells transfected with siPABPC1.

### Luciferase reporter assay

To determine the effect of PABPC1 knockdown on PSA promoter activity, siControl oligos or oligos specific for PABPC1 were transfected with PSA6.1 luc and pRL-CMV renilla plasmids into C4-2 cells using Lipofectamine 2000. After 24–48 hours cells were lysed and luciferase activity was measured on an LmaxII384 luminometer (Molecular Devices, Sunnyvale, CA) using a Promega luciferase assay kit (Promega, Sunnyvale, CA) following the manufacturer’s recommendations. The PSA6.1 firefly luciferase activity was normalized to pRL-CMV renilla luciferase activity. For hormone induction, cells were cultured in 5% charcoal stripped FBS RPMI for 24 hours followed by treatment with 0.1nM R1881 for an additional 24 hours. All experiments were done in triplicate and repeated at least three times. Significance was determined by Student’s t-test using GraphPad Prism software (San Diego, CA).

## Results

### Identification of AR^50-250^ target proteins

The NTD of the AR plays a key role in AR transactivation including androgen-independent activation of the AR in castration-resistant prostate cancer (CRPC) cells. Thus, identification of binding partners of the NTD is crucial to the design of specific therapies targeting this region. Recently, our group found that the region of amino acids 50–250 (AR^50-250^) of the NTD can promote cytoplasmic localization of the AR [12]. To determine proteins interacting with AR^50-250^, GFP pull-down was performed followed by MALDI TOF/TOF mass spectrometry analysis. MALDI TOF/TOF data was analyzed for protein identification with ABSciex Protein Pilot 3.0 using Mascot (version 2.1.0). Searches used the IPI_human_3.68 human (87061 sequences; 35160944 residues) protein database. This led to the identification of proteins as potential interacting partners with the region of a.a. 50–250 of the NTD of the AR (S1 Table). Protein scores were derived from ion scores as a non-probabilistic basis for ranking protein hits. Protein scores greater than 62 were considered to be significant (p<0.05). One protein identified was poly (A) binding protein cytoplasmic 1 (PABPC1) (S2 Table). PABPC1 was shown to have the highest score and appears to be a novel interacting partner of the AR and was thus investigated further.

### Up-regulation of PABPC1 in prostate cancer correlates to increased disease recurrence

**Fig 1:**
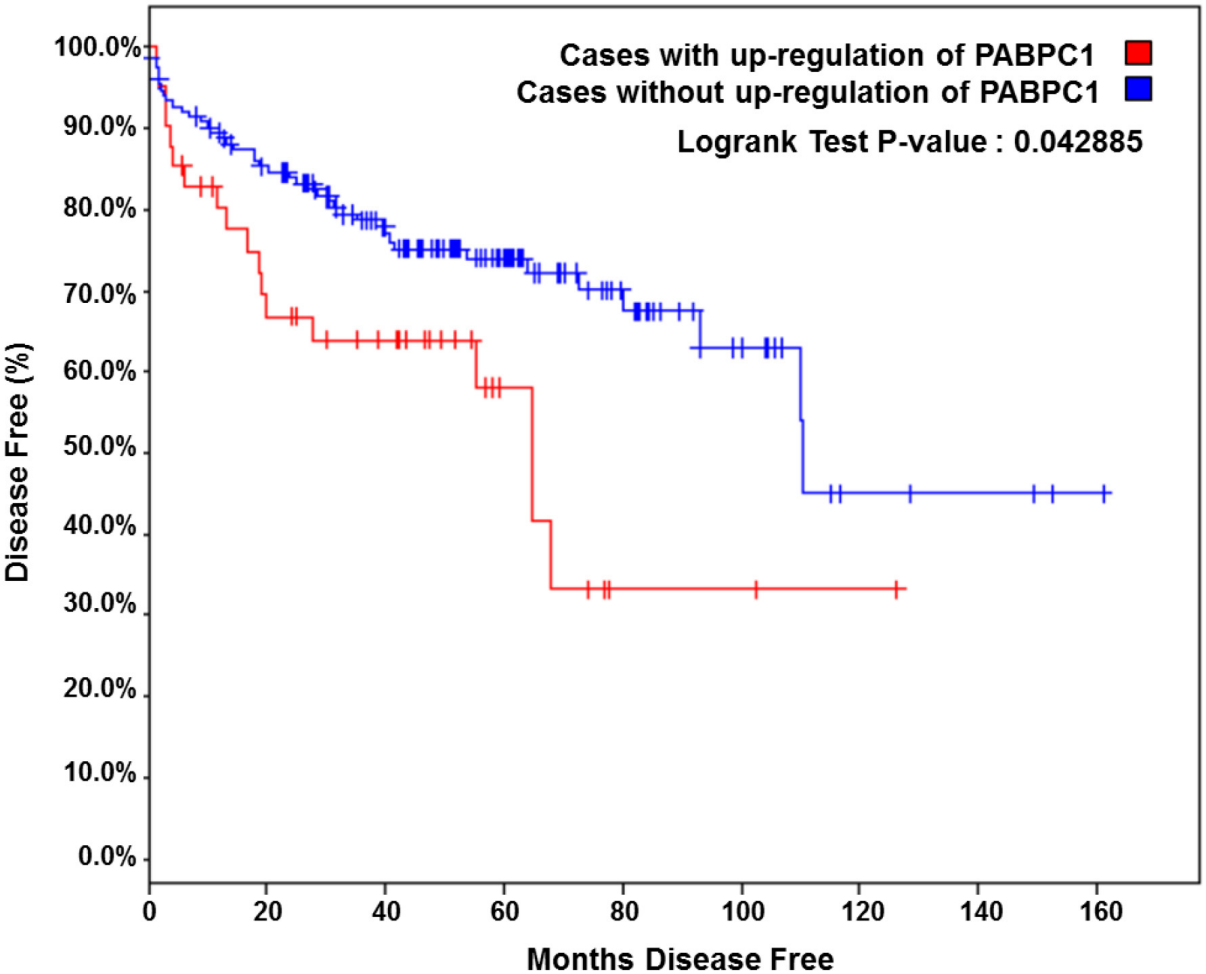
Up-regulation of PABPC1 correlates with increased disease recurrence. *In silico* analysis of the MSKCC cBioPortal for Cancer Genomics database for prostate adenocarcinoma was performed for PABPC1. Kaplan Meier plot of the risk of disease recurrence for patients with up-regulation in PABPC1 compared to patients without any alterations in PABPC1 is shown (23, 24).

To explore the expression of PABPC1 in prostate tumors, *in silico* analysis was performed using Oncomine [18]. Multiple studies [19–22] showed that PABPC1 was up-regulated in prostate cancer specimens compared with normal controls (Table 1). Additionally, analysis of the MSKCC cBioPortal for Cancer Genomics database showed that PABPC1 was up-regulated in 44 of 216 (20%) tumor samples [23–25]. In the MSKCC cohort, patients with up-regulation of PABPC1 were shown to have increased disease recurrence (Fig 1). Up-regulation of PABPC1 significantly shortened the time to disease recurrence to only 64 months disease free with up-regulated PABPC1 from 110 months disease free without increased expression of PABPC1 (Fig 1). This suggests a significant role for PABPC1 in prostate carcinogenesis.

**Table 1:** *In silico* analysis of the Oncomine database revealed that PABPC1 is up-regulated in prostate cancer tumor samples.

Research study	Study size (n)	PABPC1 up-regulation in PrCa tumors (Fold change)	p-value
Singh et al., Cancer cell 2002	235	2.288	8.92E-6
Yu et al., J Clin Oncol 2004	152	1.644	3.83E-6
Vanaja et al., Cancer Res 2003	28	1.576	2.06E-5
Luo et al., Mol Carcinog 2002	30	1.364	8.11E-4

### PABPC1 is expressed in both AR-positive and AR-negative prostate cancer cells

**Fig 2:**
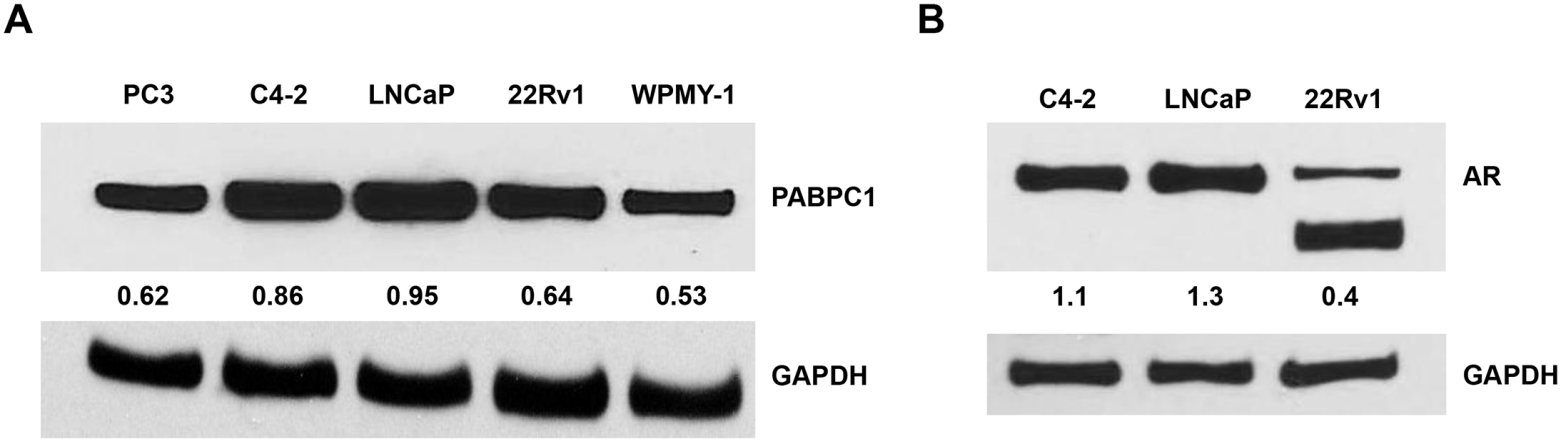
PABPC1 is expressed in prostate cancer cells. **(A)** Prostate cancer cell lines (PC3, LNCaP, C4-2, and 22Rv1) and normal prostate cells (WPMY-1) were analyzed by Western blot for protein expression levels of PABPC1 (Santa Cruz). **(B)** Expression levels of the AR in C4-2, LNCaP, and 22Rv1 cell lines. GAPDH (Santa Cruz) was used as a loading control. The numbers indicate the relative expression of PABPC1 or AR quantitated using ImageJ software when normalized to GAPDH.

Prostate cancer cells (PC3, C4-2, LNCaP, and 22Rv1) and normal prostate stromal fibroblasts (WPMY-1) were examined to determine the relative expression levels of PABPC1 (Fig 2A). Western blot analysis showed that PABPC1 is expressed in all the tested cells with slightly higher expression in AR-positive prostate cancer cells (Fig 2B).

### PABPC1 interacts with the NTD of the AR

**Fig 3:**
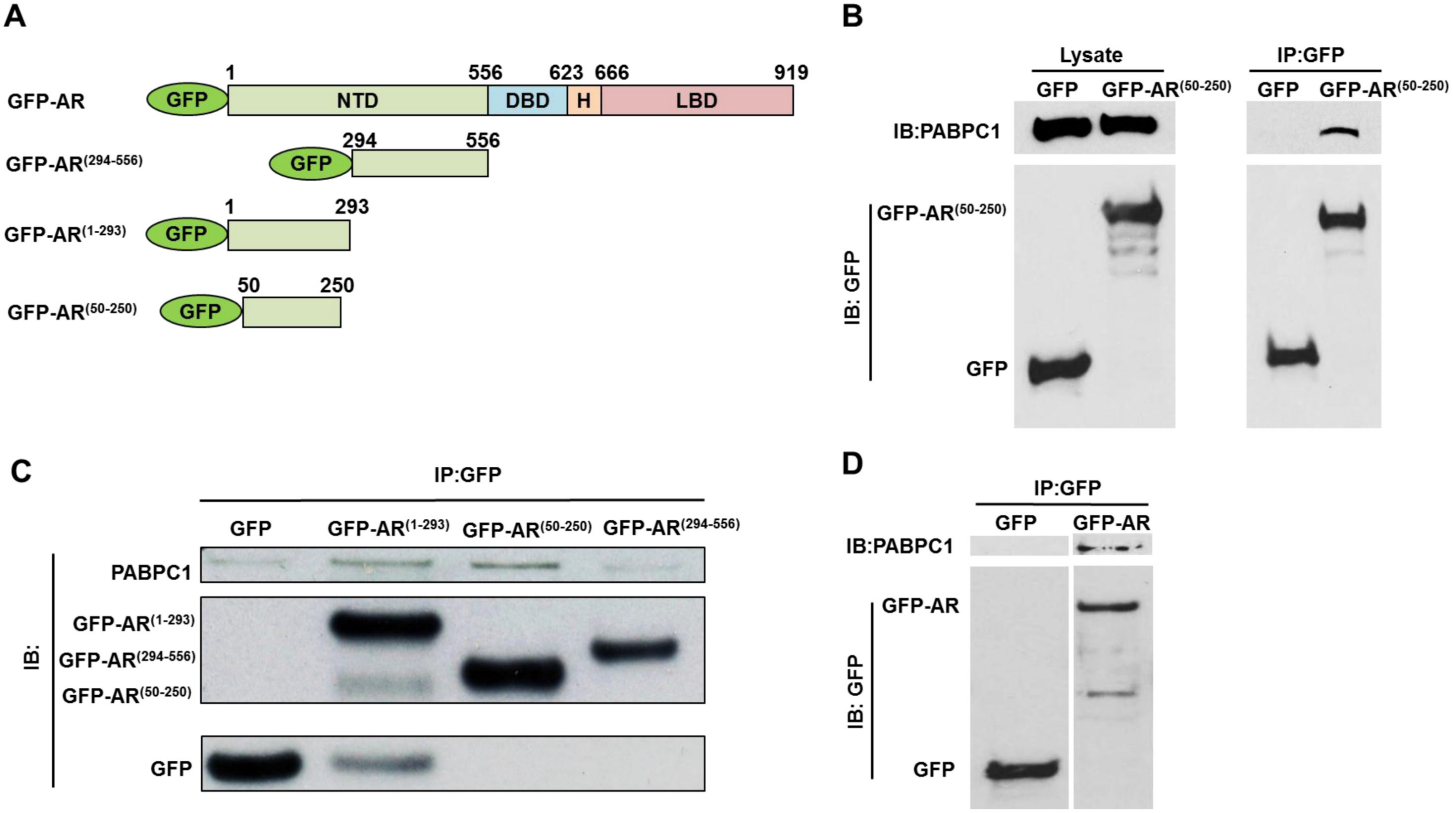
PABPC1 forms a complex with AR^50-250^ in C4-2 cells. **(A)** Schematic diagram of GFP-tagged AR (GFP-AR) and its deletion mutants, GFP-AR^(1–293)^, GFP-AR^(294–556)^ and GFP-AR^(50–250)^. The numerical numbers indicate positions of a.a. of the AR. **(B)** C4-2 cells were transiently transfected with GFP or GFP-AR^50-250^ followed by GFP pull-down. Western blot analysis was performed using antibodies specific for PABPC1 (Santa Cruz) and GFP (Torrey Pines Biolabs). **(C)** Immunoprecipitation was performed with GFP beads using C4-2 cells stably transfected with GFP, GFP-AR^(1–293)^, GFP-AR^(50–250)^, or GFP-AR^(294–556)^. Western blot analysis was performed as in (B). **(D)** GFP or GFP-AR was transiently transfected into C4-2 cells and lysates were immunoprecipitated using GFP conjugated beads followed by western blot analysis as in (B). Experiments were reproduced three times.

To confirm the interaction of PABPC1 with AR^50-250^ detected by mass spectrometry, co-immunoprecipitation was performed. C4-2 cells were transiently transfected with AR deletion mutants tagged with GFP (Fig 3A) followed by immunoprecipitation with anti-GFP beads. As shown, PABPC1 formed a complex with AR^50-250^ (Fig 3B). In addition, using C4-2 cell lines stably transfected with GFP-AR^50-250^, AR^1-293^, and AR^294-556^, it was shown that PABPC1 binds to both GFP-AR^50-250^ and GFP-AR^1-293^ (Fig 3C). To demonstrate that PABPC1 and full length AR interact, C4-2 cells were transiently transfected with GFP or GFP-AR followed by GFP pull-down assay (Fig 3D). These results suggested that PABPC1 and the AR form a complex and that PABPC1 interacts specifically with the AR^50-250^ region.

### PABPC1 knockdown inhibits AR transactivation of the androgen responsive gene PSA

**Fig 4:**
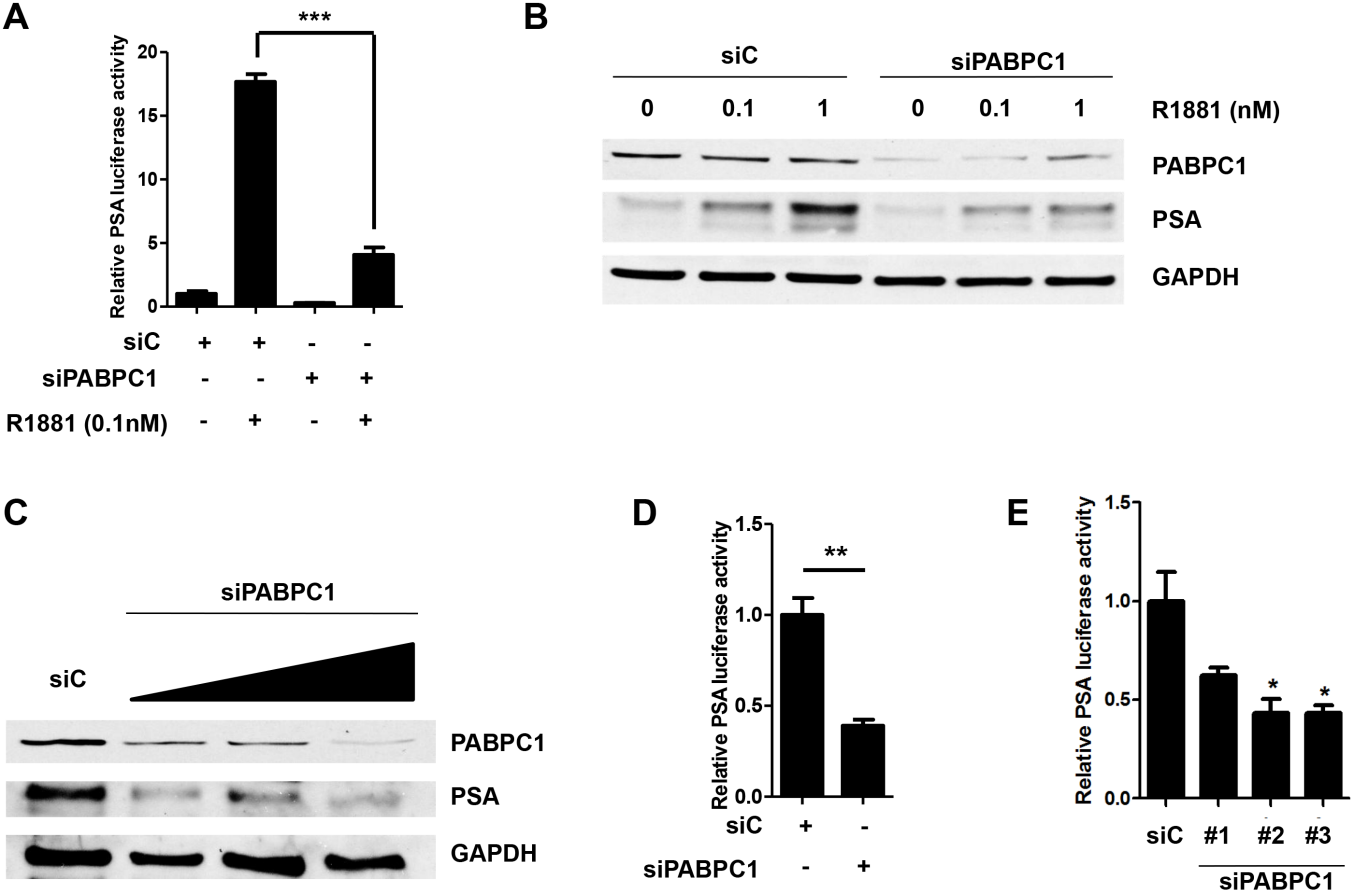
Knockdown of PABPC1 with siRNA decreases PSA luciferase activity and PSA protein levels. **(A)** C4-2 cells were transfected with control siRNA (siC) (40 pmol/mL), siPABPC1 (pool of 3 oligos) (40 pmol/mL), pRL-CMV (0.03μg), and PSA6.1Luc (0.3μg) in OPTI-MEM. The media was changed the next day to 5% chS FBS RPMI for 24 hours followed by treatment with 0.1nM R1881for an additional 24 hours in 5% chS FBS RPMI. Luciferase assay was performed with pRL-CMV (Renilla) luciferase used as a normalizer. **(B)** C4-2 cells were transfected with control siRNA (siC) or siPABPC1 (40 pmol/mL) and treated as in (A) using increasing doses of R1881 (0, 0.1, and 1nM) followed by Western blot analysis. Blots were probed with antibodies specific for PABPC1 and PSA. GAPDH was used as a loading control. **(C)** C4-2 cells were transfected with control siRNA (siC) or siPABPC1 (pool of 3 oligos) (20 pmol/mL, 40 pmol/mL and 80 pmol/mL), in OPTI-MEM. Media was changed the next day to 10% FBS RPMI for 72 hours followed by Western blot analysis. Blots were probed as in (B). C4-2 cells were transfected with control siRNA (siC) or siPABPC1 (pool of 3 oligos) (40 pmol/mL), **(D)** or individual PABPC1 siRNA oligos **(E)**, pRL-CMV (0.03μg), and PSA6.1Luc (0.3μg) in OPTI-MEM. The media was changed the next day to 10% FBS RPMI for an additional 48 hours followed by luciferase assay. Experiments were repeated three times. Significance was determined by Student’s t-test (*p<0.05, **p<0.01, ***p<0.001).

The AR is a ligand dependent transcription factor known to bind to androgen response elements (AREs) and activate transcription of androgen responsive genes such as PSA [26], Nkx3.1[27], and VEGF [28]. Since PABPC1 was shown to interact with the AR, we next examined the effects of knockdown of PABPC1 on AR transactivation using siRNA. C4-2 cells were transfected with control siRNA or siRNA (pool of three oligos) specific for PABPC1. Knock down of PABPC1 significantly suppressed androgen induction of PSA luciferase activity more than 80% compared to control siRNA (Fig 4A). This was confirmed by western blot analysis, which also showed inhibition of androgen induced PSA protein levels when PABPC1 was knocked down (Fig 4B). Additionally, knock down of PABPC1 suppressed PSA protein levels and luciferase in complete media (10% FBS RPMI) (Fig 4C and 4D). Similar results were seen with the individual oligos from the pool of PABPC1 siRNA oligos, indicating that this effect was not due to non-specific targeting of the siRNAs (Fig 4E). These results suggested that PABPC1 is a novel co-regulator of the AR in prostate cancer cells.

### Knockdown of PABPC1 inhibits activation of the PSA promoter by NAR (AR lacking LBD)

**Fig 5:**
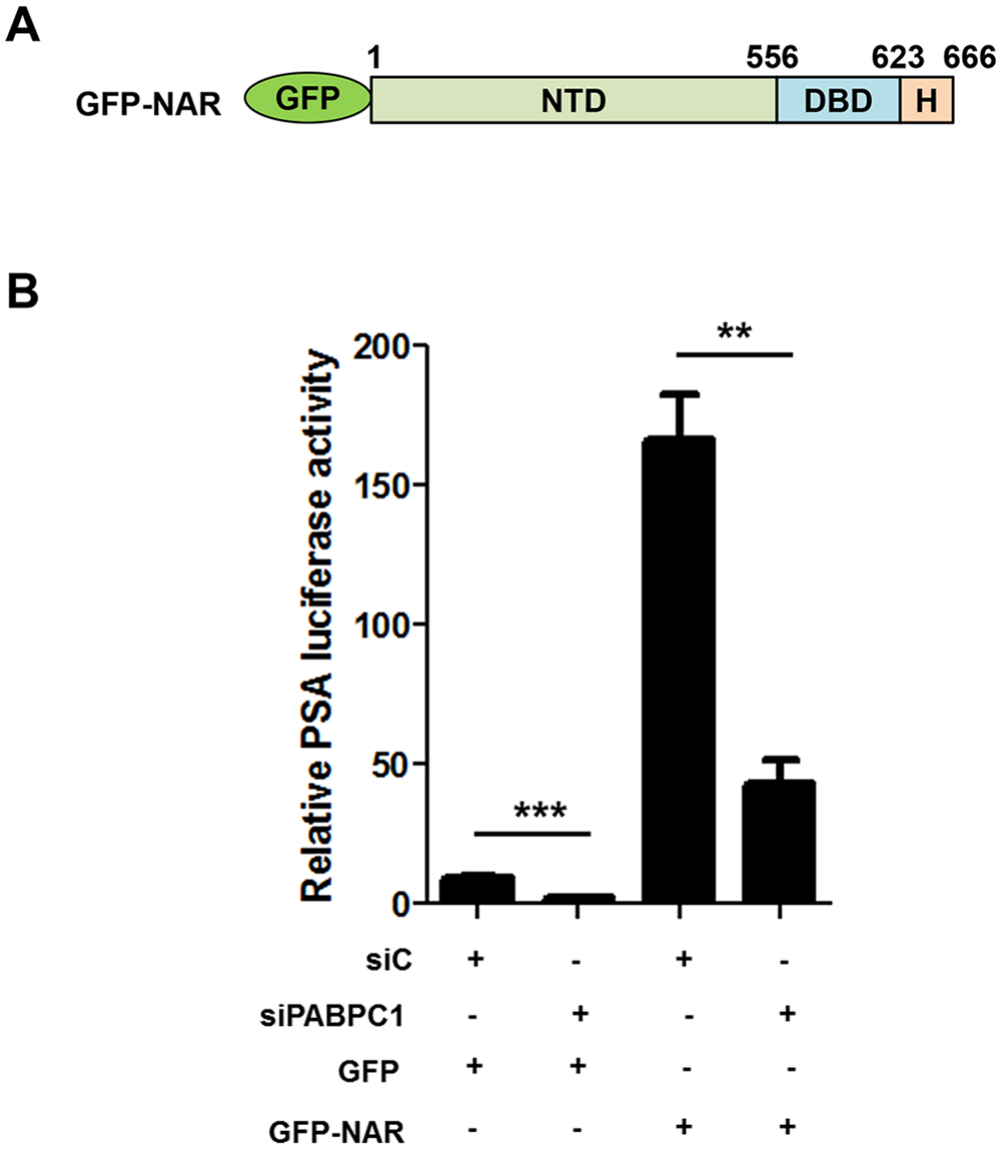
Knockdown of PABPC1 inhibits activation of the PSA promoter by NAR. **(A)** Schematic diagram of GFP-NAR. **(B)** C4-2 cells were transfected with control siRNA (siC) or siPABPC1 (pool of 3 oligos) (40 pmol/mL) for 48 hours followed by transfection with PSA6.1Luc, pRL-CMV, and either GFP (control) or GFP-NAR in the presence of 5% charcoal stripped FBS RPMI for an additional 48 hours followed by luciferase assay. Experiments were repeated two times. Significance was determined by Student’s t-test (**p<0.01, ***p<0.001).

Recently, splice variants of the androgen receptor lacking the LBD have been shown to activate transcription of androgen response genes [29,30]. Here, an AR mutant construct lacking the entire LBD region, NAR, (Fig 5A) was transfected into C4-2 cells that were pretreated with siRNA specific for PABPC1 to determine if PABPC1 could regulate the up-regulation of androgen response genes by AR variants. Indeed, this was the case as knockdown of PABPC1 vastly decreased the activation of the PSA promoter (Fig 5B). This result along with the previous result of PABPC1 interacting with the NTD of the AR, suggests that PABPC1 could be binding to the NTD of the AR and blocking activation of androgen response genes through inhibition of the AR and its splice forms lacking the LBD.

### Reduction of nuclear AR by PABPC1 knockdown

**Fig 6:**
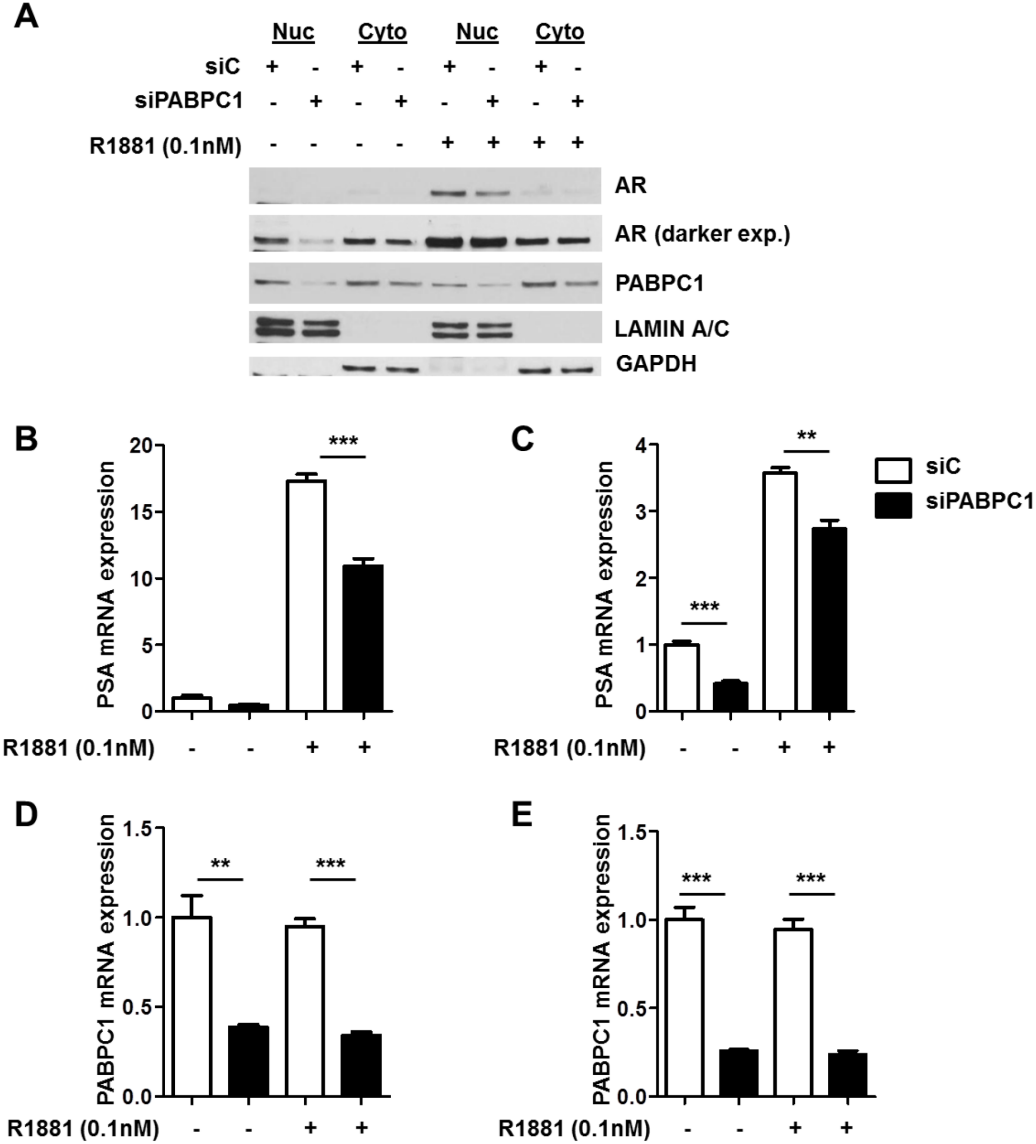
Knockdown of PABPC1 with siRNA decreases nuclear AR protein levels and PSA mRNA expression levels. **(A)** C4-2 cells were transfected with control siRNA (siC) (40 pmol/mL) or siRNA (40 pmol/mL) specific for PABPC1 (siPABPC1) for 48 hours followed by treatment with 0.1nM R1881 for 24 hours in the presence of 5% charcoal-stripped FBS RPMI. Nuclear and cytoplasmic extracts were analyzed by Western blot. GAPDH (Santa Cruz) and Lamin A/C (Genscript, Piscataway, NJ) were used as loading and cell compartment controls. PSA **(B-C)** and PABPC1 **(D-E)** mRNA expression levels were detected in LNCaP and C4-2 cells following siPABPC1 treatment as in (A). Expression was normalized to GAPDH. Experiments were reproduced twice. Significance was determined by Student’s t-test (**p<0.01, ***p<0.001).

To determine the effect of inhibition of PABPC1 expression on AR nucleocytoplasmic trafficking, C4-2 cells were transfected with siPABPC1 for 48 hours followed by treatment with 0.1nM R1881 or vehicle for an additional 24 hours. Western blot analysis of nuclear and cytoplasmic fractions revealed that nuclear AR protein levels were reduced with the knockdown of PABPC1 in C4-2 cells (Fig 6A). Additionally, hormone induction of PSA mRNA levels in both LNCaP (Fig 6B) and C4-2 cells (Fig 6C) was significantly reduced by knockdown of PABPC1 (Fig 6D and 6E). Another androgen response gene, FKBP5, was tested for specificity of PABPC1 to androgen response gene expression. Inhibition of PABPC1 was shown to decrease the androgen up-regulation of FKBP5 by R1881 (S1 Fig). These results suggest that PABPC1 controls the amount of the AR in the nucleus and plays a role in the transactivation of AR target genes in these cell lines.

### Suppression of PABPC1 inhibits AR-positive prostate cancer cell proliferation

**Fig 7:**
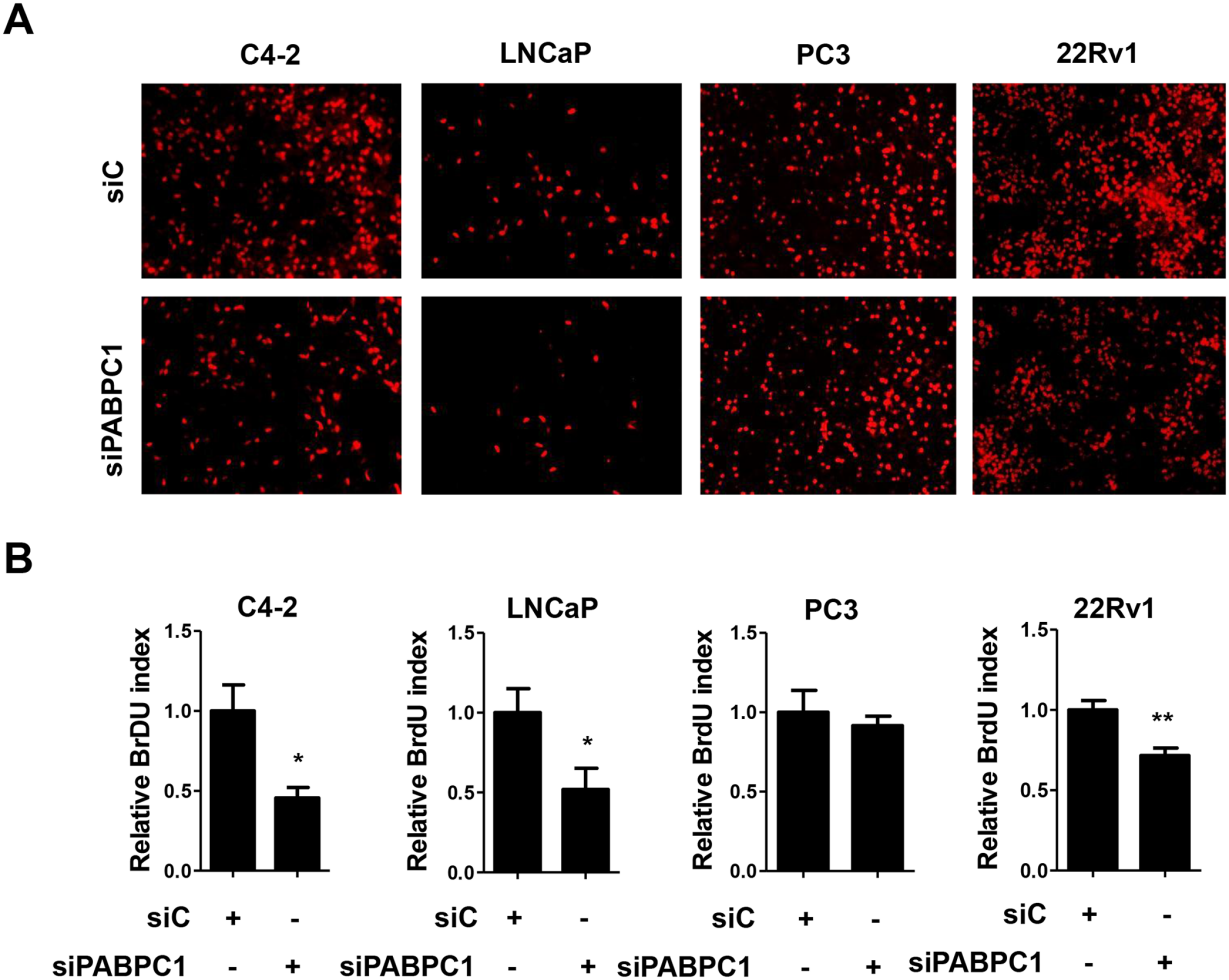
Inhibition of PABPC1 decreases proliferation of AR positive prostate cancer cells. C4-2, LNCaP, PC3, and 22Rv1 cells were transfected with control siRNA (40 pmol/mL) or a pool of siPABPC1 oligos (40 pmol/mL) followed by BrdU assay (as described in the Methods section). **(A)** Shown are representative images from three individual experiments of cells treated with either control siRNA (siC) or siPABPC1. **(B)** BrdU positive cells were counted and compared between siC and siPABPC1 groups. Experiments were reproduced three times. Significance was determined by Student’s t-test. (*p<0.05, **p<0.01).

To determine the effect of PABPC1 on prostate cancer cell proliferation, siRNA specific for PABPC1 knockdown was transfected into C4-2, LNCaP, PC3, and 22Rv1 cells. Knockdown efficiency was determined by Western blot (S2 Fig). AR-positive C4-2, LNCaP, and 22Rv1 cells exhibited significantly less proliferation upon PABPC1 knockdown when compared to control cells (Fig 7). In contrast, AR-negative PC3 cell proliferation was not affected by PABPC1 knockdown. This suggested that PABPC1 plays a role in the proliferation of AR-positive prostate cancer cells including castration-resistant C4-2 cells.

## Discussion

The AR plays a major role in all stages of prostate cancer development and progression, including CRPC, thus it is important to determine the mechanisms involved including the impact of AR co-regulators. This study identified PABPC1 as a novel AR co-regulator capable of modulating AR function and subcellular localization in prostate cancer cells. PABPC1 is associated with the AR through the NTD region (AR^50-250^) that can promote AR cytoplasmic localization. Knockdown of PABPC1 decreased AR transcriptional activity and also reduced nuclear AR in C4-2 prostate cancer cells. Furthermore, PABPC1 knockdown inhibited proliferation of all the tested AR-positive but not the AR-negative PC3 prostate cancer cells. *In silico* analysis of several publicly available datasets including the MSKCC cBioPortal for Cancer Genomics database revealed PABPC1 up-regulation in prostate cancer specimens and this up-regulation was associated with rapid disease recurrence. These findings suggest a significant role for PABPC1 in modulating AR activity and prostate carcinogenesis.

PABPC1 appears to play important roles in AR function in both androgen-sensitive and castration-resistant prostate cancer cells. In both androgen-sensitive LNCaP and castration-resistant C4-2 cells, PABPC1 knockdown reduced PSA-based luciferase activity as well as inhibited the expression of endogenous PSA mRNA and protein. These findings suggest that PABPC1 up-regulation in prostate cancer cells can enhance androgen signaling and subsequently promote prostate cancer cell proliferation in both androgen-sensitive and castration-resistant stages of the disease.

PABPC1 appears to inhibit AR nuclear export and/or enhance AR nuclear import, because PABPC1 knockdown decreased endogenous nuclear AR protein levels in C4-2 cells. The modulation of AR subcellular localization by PABPC1 is likely mediated through AR^50-250^ (a region in the NTD capable of promoting AR cytoplasmic localization) because PABPC1 is associated with this region in co-immunoprecipitation assays. Since AR^50-250^ promotes AR cytoplasmic localization using the CRM-1-dependent nuclear export pathway [12], the modulation of AR subcellular localization by PABPC1 is likely mediated through this pathway as well. Our finding also indicates that AR^50-250^ in the context of the full-length AR can modulate intracellular trafficking via PABPC1 because PABPC1 association with AR is specifically mediated through AR^50-250^.

PABPC1 modulation of the AR is likely to have a significant impact on prostate cancer progression because the AR is a key factor regulating prostate cancer cell growth and factors capable of modulating AR function could have profound effects in modulating prostate cancer cell growth. We recognize that PABPC1 has the potential to modulate multiple signaling pathways. As a poly A binding protein, it could influence translation of multiple signaling molecules. However, its influence on prostate cancer cell growth is likely mediated through the AR because PABPC1 knockdown inhibited proliferation of AR-positive prostate cancer cells, but not AR-negative PC3 cells. The up-regulated expression of PABPC1 in prostate cancer specimens and the association of high-level PABPC1 expression with recurrent prostate cancer further suggest that PABPC1 plays an important role in promoting prostate cancer progression.

Identification of PABPC1 as a co-factor of the AR provides a potential link between AR activity and active translation in prostate cancer cells. PABPC1 is thought to stimulate efficient mRNA cap-dependent translation. Thus, elevated PABPC1 expression levels in prostate cancer cells can simultaneously enhance translation and androgen action, which may lead to more efficient cell growth. As a CRM-1 dependent shuttling protein between nuclear and cytoplasmic regions [31,32], PABPC1 may modulate AR activity via regulating the nucleocytoplasmic trafficking of the AR. However, PABPC1 may influence AR activity via interaction with other co-regulators of the AR. For example, PABPC1 can interact with paxillin [31,32], which is also an AR co-factor [33]. Paxillin is capable of modulating AR nucleocytoplasmic trafficking [33], suggesting that paxillin may facilitate or modulate the PABPC1 regulation of the AR. Though, we cannot rule out the possibility that PABPC1 can modulate the stability of AR in the nucleus.

In summary, the results of our present study suggest that PABPC1 is an important AR co-regulator capable of promoting AR nuclear localization and function via interaction with the NTD region. Elevated expression of PABPC1 in prostate cancer specimens is likely to play an important role in prostate carcinogenesis by enhancing AR signaling pathways. Future studies aimed at defining the precise mechanism of PABPC1 regulation of AR signaling may lead to new approaches to prevent and/or treat prostate cancer patients.

## Supporting Information

S1 Fig Androgen induction of FKBP5 is reduced by inhibition of PABPC1. cDNA lysates used in Fig 6 were assayed for expression of FKBP5 mRNA levels by real-time PCR.
(TIF)

S2 Fig Efficiency of PABPC1 knockdown in prostate cancer cell lines. C4-2, LNCaP, 22Rv1, and PC3 cells were transfected with siC or siPABPC1 for 72 hours followed by Western blot analysis. Antibodies specific for PABPC1 and GAPDH were used to probe the blots as previously described.
(TIF)

S1 Table Identification of interacting partners of a.a. 50–250 of the AR by mass spectrometry. (TIF)

S2 Table PABPC1 peptide bands interacting with a.a. 50–250 of the AR. (TIF)
