# Supplementary figures and images for: Poly (A) Binding Protein Cytoplasmic 1 Is a Novel Co-Regulator of the Androgen Receptor
Source: PLoS One. 2015 Jul 15;10(7):e0128495. doi: 10.1371/journal.pone.0128495 (PMC4503479; doi:10.1371/journal.pone.0128495)

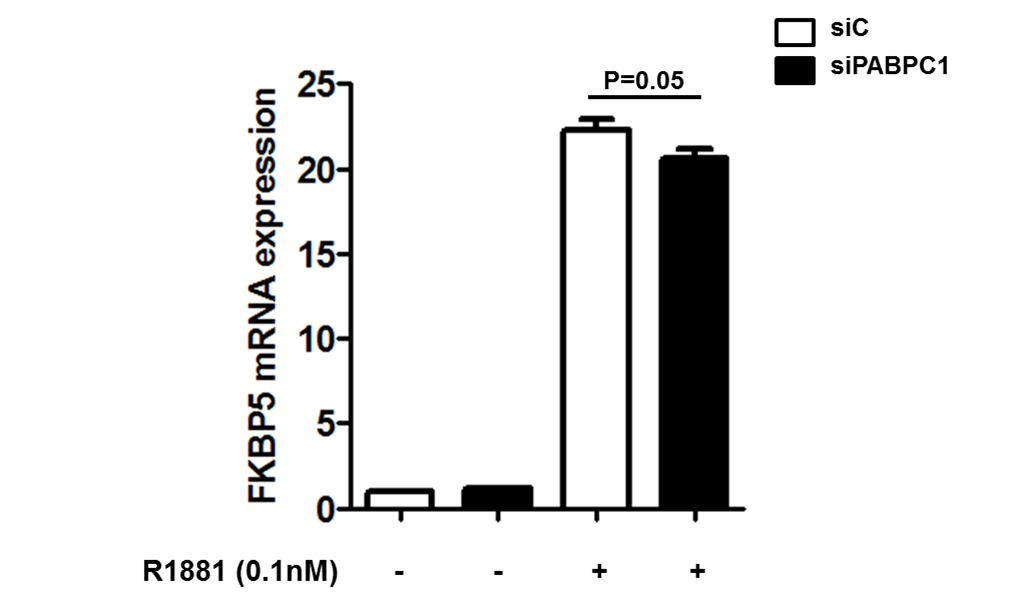

Supplement: S1 Fig — cDNA lysates used in Fig 6 were assayed for expression of FKBP5 mRNA levels by real-time PCR. (TIF) [file pone.0128495.s001.tif]

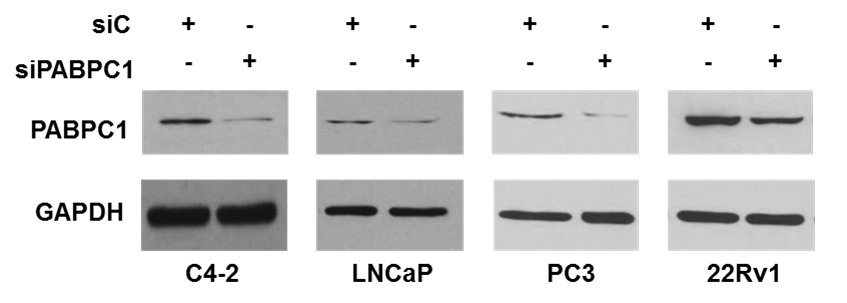

Supplement: S2 Fig — C4-2, LNCaP, 22Rv1, and PC3 cells were transfected with siC or siPABPC1 for 72 hours followed by Western blot analysis. Antibodies specific for PABPC1 and GAPDH were used to probe the blots as previously described. (TIF) [file pone.0128495.s002.tif]

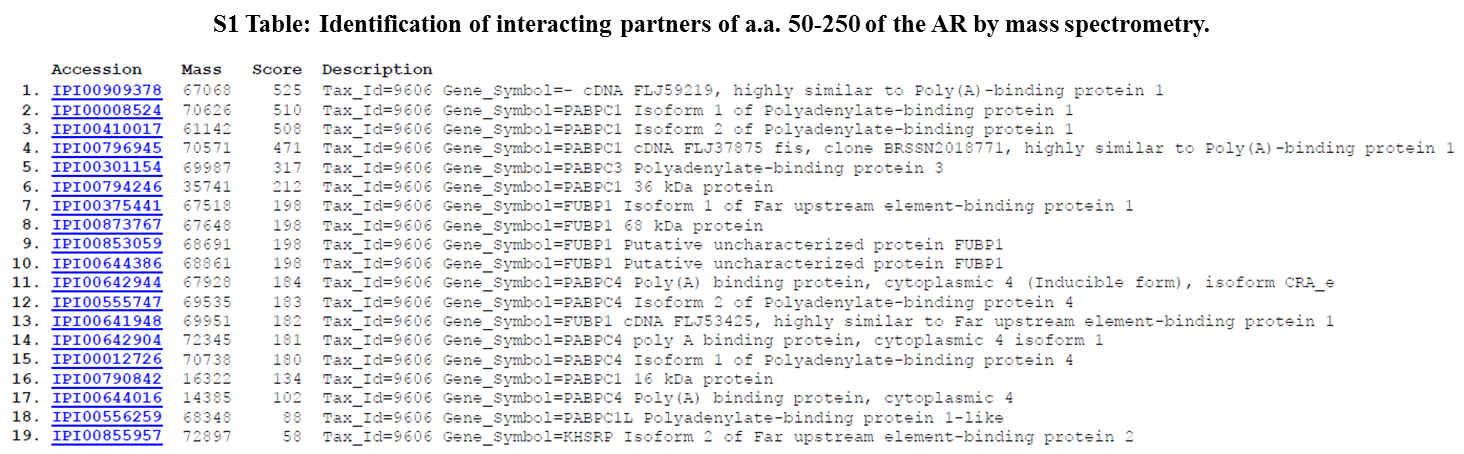

Supplement: S1 Table — (TIF) [file pone.0128495.s003.tif]

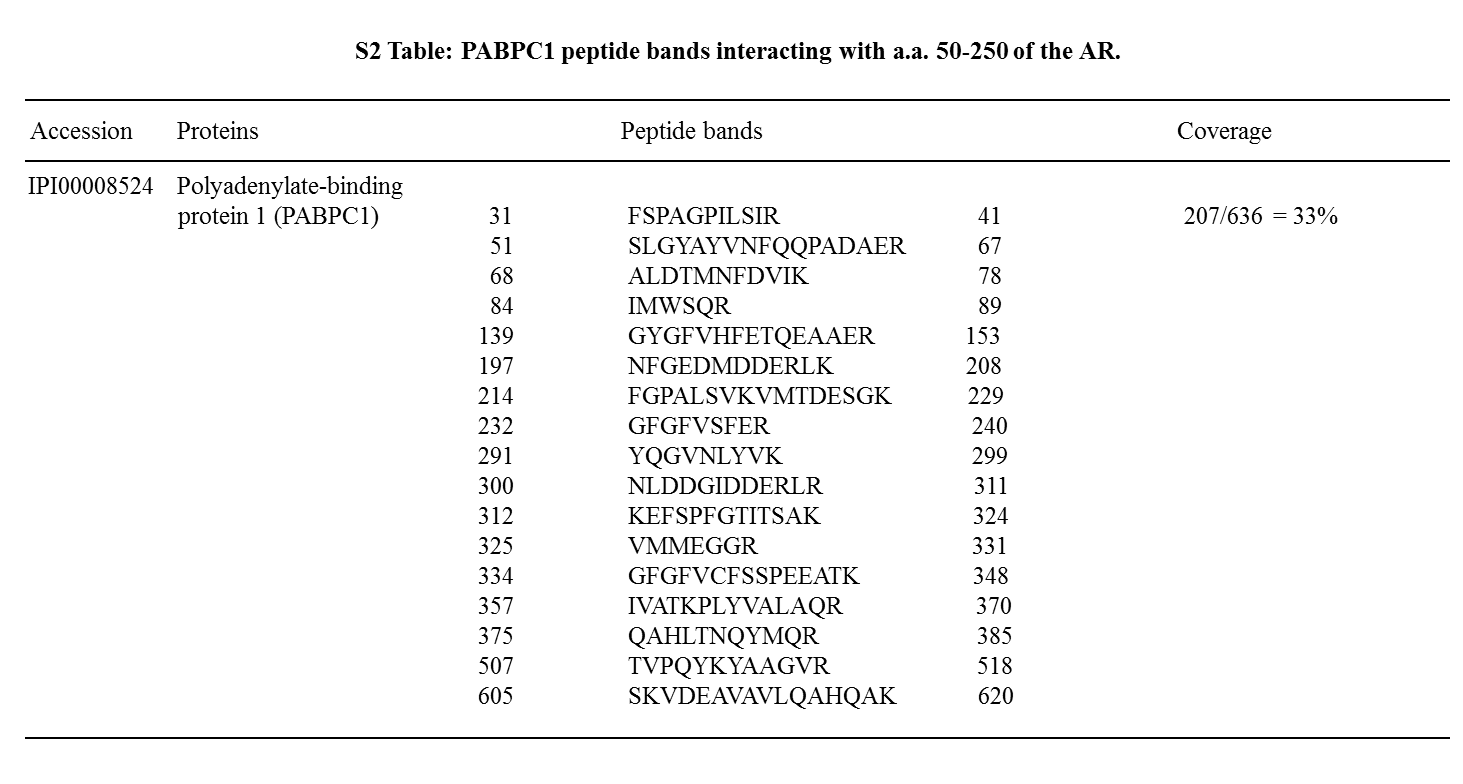

Supplement: S2 Table — (TIF) [file pone.0128495.s004.tif]
